# Pore-Mouth Structure of Highly Agglomerated Detonation Nanodiamonds

**DOI:** 10.3390/nano11112772

**Published:** 2021-10-20

**Authors:** Elda Zoraida Piña-Salazar, Kento Sagisaka, Takuya Hayashi, Yoshiyuki Hattori, Toshio Sakai, Eiji Ōsawa, Katsumi Kaneko

**Affiliations:** 1 Research Initiative for Supra-Materials, Interdisciplinary Cluster for Cutting Edge Research, Shinshu University, Nagano 380-8553, Japan; eldazps@shinshu-u.ac.jp (E.Z.P.-S.); hayashi@endomoribu.shinshu-u.ac.jp (T.H.); hattoriy@shinshu-u.ac.jp (Y.H.); 2 Division of Chemistry and Materials, Faculty of Textile Science and Technology, Shinshu University, Ueda 386-8567, Japan; 16st302j@shinshu-u.ac.jp; 3 Department of Materials Chemistry, Faculty of Engineering, Shinshu University, Nagano 380-8553, Japan; tsakai@shinshu-u.ac.jp; 4 Nano-Carbon Research Institute, Ltd., Ueda 386-8567, Japan; osawa@nano-carbon.jp

**Keywords:** nanodiamonds, long-heated, agglomeration, pore-mouth structure, water adsorption

## Abstract

Detonation nanodiamond aggregates contain water that is removed by thermal treatments in vacuo, leaving available pores for the adsorption of target molecules. A hard hydrogel of detonation nanodiamonds was thermally treated at 423 K for 2 h, 10 h, and 52 h in vacuo to determine the intensive water adsorption sites and clarify the hygroscopic nature of nanodiamonds. Nanodiamond aggregates heated for long periods in vacuo agglomerate due to the removal of structural water molecules through the shrinkage and/or collapse of the pores. The agglomerated nanodiamond structure that results from long heating periods decreases the nitrogen adsorption but increases the water adsorption by 40%. Nanodiamonds heated for long times possess ultramicropores <0.4 nm in diameter in which only water molecules can be adsorbed, and the characteristic mouth-shaped mesopores adsorb 60% more water than nitrogen. The pore mouth controls the adsorption in the mesopores. Long-term dehydration partially distorts the pore mouth, decreasing the nitrogen adsorption. Furthermore, the nitrogen adsorbed at the pore mouth suppresses additional nitrogen adsorption. Consequently, the mesopores are not fully accessible to nitrogen molecules because the pore entrances are blocked by polar groups. Thus, mildly oxidized detonation nanodiamond particles can show a unique molecular sieving behavior.

## 1. Introduction

Currently, there is an increasing need for studying and controlling the properties of new materials used as adsorbents and catalysis to use them in green routes. In this sense, water was used to manipulate the catalytic activity of biomass char through exposure to steam with the aim of hydrogen production [1]. Among the variety of adsorbents, materials with a pore mouth structure are relevant for their potential to confine water and other molecules of interest. For example, the confinement of a cyclic complex molecule at the pore mouth structure of a zeotype was reported to study its interaction with the pore mouth structure, which becomes relevant from the adsorption point of view [2]. The control of humidity levels is important to assure human comfort and the proper state of certain materials, equipment, goods, food, etc. Hygroscopic materials are used for humidity control due to their ability to quickly absorb water. There exist materials that can adsorb water, such as carbon blacks, clays, etc. Nanocarbons are the most common and abundant adsorbents due to their stability and high adsorption properties. Activated carbon yields great water adsorption, carbon nanofibers can be synthesized to yield mesoporosity and work as adsorbents, graphene oxide has the potential to hydrate; however, it tends to aggregate. The unique core–shell structure of nanodiamonds has ultramicropores in the interstices of the aggregated particles and the core–shell structure, and the graphitic carbon shell is potentially modifiable. In addition, their non-toxicity and biocompatibility make them attractive for biological uses such as drug delivery, high adsorption of water-soluble vitamins [3], and cell therapies as hydrogels [4], taking advantage of their high hygroscopic nature. They are also attractive for water purification and wastewater treatment [5], and they are also used as sorbents in chromatography and lubricants. Aside from the aforementioned potential uses of nanodiamonds, aggregates of nanodiamonds can be hydrated and retain moisture when exposed to humid air [6,7]. This property originates from the formation of strong hydrogen bonds due to electron accumulation at the nanodiamond–water interface in hydrogenated nanodiamonds [8]. Recently, we reported the relationship between the pore structure of nanodiamond aggregates and water vapor adsorption. The higher the outgassing temperature of nanodiamonds, the greater their ability to adsorb water [9]. Nanodiamond particles have a core structure of sp^3^-hybridized carbon covered by partially oxidized sp^2^-hybridized carbon surrounded by a hydration shell. Hard hydrogels of nanodiamonds possess a hierarchical porosity of micropores and mesopores and a high specific area of approximately 300 m^2^ g^−1^ after being heated at 623 K for 2 h in vacuo [7]. Ji et al. reported the strong adsorption of water molecules even at a relative humidity (RH) lower than 35% [6]. Water contained in nanodiamonds can be removed by long-term thermal treatments in vacuo, such as 423 K for 15 h under high vacuo [10] or at 473 K under 15 hPa (10^3^ Pa) for 10 h for the determination of water contents in nanodiamond gels (stable gels of extremely small diamond crystals 2 nm in diameter) [11]. The removal of tightly bound water produces a large number of pores that are available for the further adsorption of water or target molecules. The hydrophilic properties of materials can be evaluated by measuring the water adsorption under different relative humidities [12,13]. However, an exact understanding of the water adsorption nature of nanodiamonds can be obtained by water adsorption measurements ranging from in vacuo to a relative pressure (P/P_0_) ≈ 1. The adsorption of water on a nanodiamond film has been explained by the classical model for the adsorption of water on oxygen-containing surface groups [14]. Our previous research clarified the water affinity of nanodiamonds for hydrophilic sites in the nanodiamond assembly due to the presence of small micropores [7]. Nanodiamonds are suitable for applications that involve dispersion media [15,16,17]. Therefore, we need to elucidate the water-adsorbed state in the pore structure of nanodiamonds. Probe molecules nitrogen, argon, and water, among others, have been used to define the pore structure of porous adsorbent materials [18,19]. In the present study, we reveal the pore structure changes in nanodiamonds due to long-term heat treatment in vacuo by measurements of argon, nitrogen, and water adsorption and correlate them to water adsorptivity.

## 2. Materials and Methods

### 2.1. Sample Preparation

Nanodiamonds in the form of a hard hydrogel (Nano-Carbon Research Institute, Ltd., Ueda, Japan) were outgassed at 393 K for 2 h in a vacuum oven. Afterward, the sample was separated into several portions to be thermally treated at 423 K and 623 K for 2 h up to 52 h.

### 2.2. Surface Analysis of Nanodiamonds

Thermogravimetric analysis from room temperature to 1300 K of nanodiamonds before and after heating at 423 K for 2 h and 52 h in an Ar atmosphere (100 mL min^−1^) was performed by using a thermal analyzer (STA 7200 Thermal Analysis System, Hitachi High-Tech Science Corp., Tokyo, Japan). X-ray photoelectron spectroscopy (XPS) was carried out to examine the surface chemistry of nanodiamonds before and after the thermal treatments in vacuo by using an XPS instrument (Kratos Axis Ultra X-ray Photoelectron Spectroscopy system, Shimadzu Co., Kyoto, Japan) with an Al K**α** X-ray 12 kV source. Heated samples were maintained in vacuo without exposure to air before the thermogravimetric and XPS analyses. The obtained XPS results were calibrated at 285 ± 0.2 eV, corresponding to the sp^3^ carbon of nanodiamond. The X-ray diffraction (XRD) pattern of nanodiamonds was obtained at room temperature using an X-ray diffractometer (SmartLab X-ray, Rigaku Co., Tokyo, Japan) with a Cu K**α** (40 kV and 30 mA) source. HRTEM images of dry nanodiamonds deposited on a TEM grid were taken using a High-Resolution Transmission Electron Microscope (HR-TEM; JEOL, JEM-2100F, JEOL Ltd., Tokyo, Japan) operated at 200 kV. For comparison, samples of nanodiamonds heated at 623 K for 2 h, 10 h and 52 h in vacuo were also analyzed.

### 2.3. Adsorption of Nitrogen at 77 K, Argon at 87 K, and Water at 298 K on Nanodiamonds

The pore structure of nanodiamonds was evaluated by nitrogen adsorption isotherms at 77 K. Samples of nanodiamonds after outgassing at 423 K for 2 h, 10 h, or 52 h were measured using a volumetric adsorption apparatus (Quantachrome-Autosorb IQ2, Anton Paar, Graz, Austria) to evaluate the porosity. Pore size distributions were determined by quenched solid density functional theory (QS–DFT) assuming a slit-shaped pore geometry model. Water adsorption isotherms were obtained at 298 K; the nanodiamonds were outgassed at 423 K for 2 h, 10 h, and 52 h, and the measurements were performed on a VSTAR instrument (Quantachrome, Anton Paar, Graz, Austria).

## 3. Results

### 3.1. Chemical and Structural Changes of Nanodiamond Aggregates upon Heating

**Figure 1:**
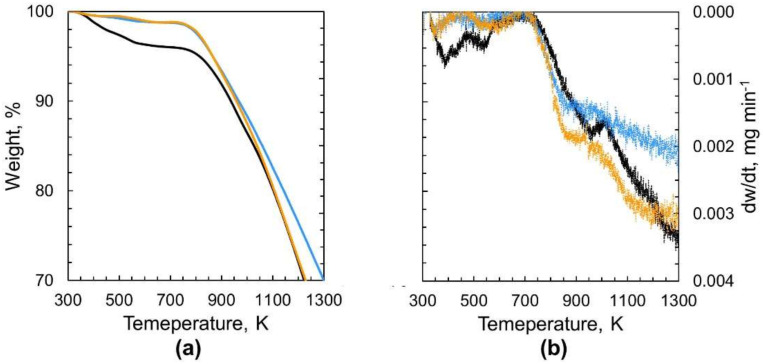
(**a**) Thermogravimetric (TG) and (**b**) TG differential profiles of nonthermally treated nanodiamonds (black line **−**) and nanodiamonds heated at 423 K for 2 h (light blue line **−**) and 52 h (orange line **−**) under an argon atmosphere (Ar 100 mL min^−1^).

Thermogravimetric (TG) profiles of nanodiamonds before and after heating at 423 K for 2 h and 52 h are shown with their derivative profiles in Figure 1. The TG plot clearly shows the substantial weight loss of the nonthermally treated nanodiamonds below 750 K that is higher than the weight loss observed for nanodiamonds heated at 423 K. This weight loss is associated with the removal of the water contained in nonthermally treated nanodiamonds. The nanodiamond hydrogel contains water that can be removed by heating at 473 K for 10 h [11]. Weakly adsorbed water desorbs from nanodiamonds below 373 K, while strongly adsorbed water desorbs between 373 and 573 K [20]. For comparison, the profiles of thermogravimetric analysis of nanodiamonds heated at 623 K for long periods of 2 h and 52 h are presented in Figure S1. Nanodiamond samples were stored under an argon atmosphere after heating at the target temperature and time before the thermogravimetric analysis was performed from room temperature to 1300 K. Therefore, the difference in weight loss below 700 K observed between nonheated and heated nanodiamonds must be mainly ascribed to the removal of water molecules strongly bound in the nanodiamond samples, as shown in Figure 1. The weight loss of nanodiamonds heated at 423 K for 2 h above 1000 K is larger than that of nanodiamonds heated at 423 K for 52 h. This result indicates the presence of strongly bound water in the nanodiamonds.

**Figure 2:**
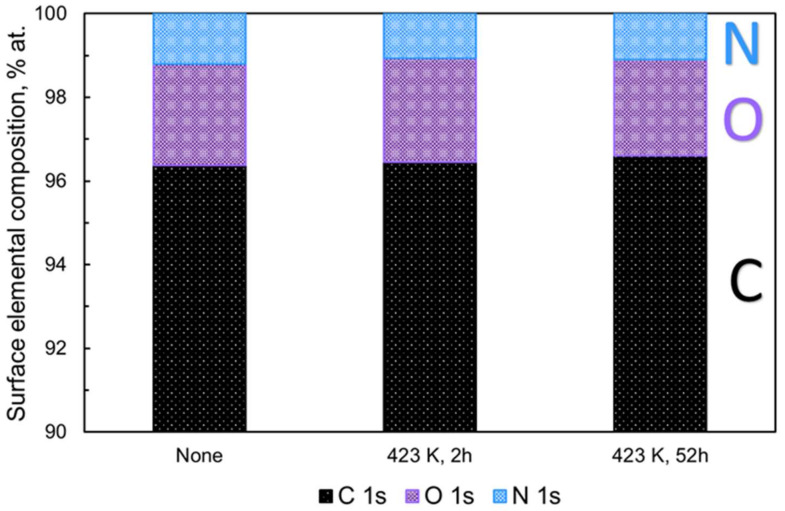
Atomic contents (at %) of C 1 s, O 1 s, and N 1 s of nonheated and heated nanodiamonds at 423 K for 2 h and 52 h from XPS analysis.

Figure 2 shows the atomic percent (at %) of carbon (C 1s), oxygen (O 1s), and nitrogen (N 1s) in nanodiamonds determined by XPS before and after thermal treatments at 423 K for 2 h and 52 h. The contents of the C 1s, O 1s, and N 1s core levels are 96.5 at %, 2.5 at %, and 1.0 at %, respectively, with an O/C ratio of 2.6 × 10^−2^ before heating in vacuo.

The XPS analysis shows no significant surface composition change in the nanodiamonds regardless of the heat treatment; the variations are below 0.2 at %, as shown in Figure 2 and Figure S2. In addition, Figure S2 and Table 1 show the carbon constituents of the nanodiamond before and after heating in vacuo derived from the deconvolution of the high-resolution C 1s spectra; the presence of a slight amount of carbon bonded to oxygen and the co-presence of C-N, C-H, and C-OH at a binding energy of 286.2 eV are observed. The major constituent of the nanodiamonds is sp^3^-hybridized carbon, which is followed by sp^2^-hybridized carbon, C bonded to hydrogen, nitrogen, and hydroxyl groups, and finally C-O corresponding to oxygen-containing functionalities situated on the graphene-like carbon shell surrounding the diamond core. We observe a decrease in sp^2^-hybridized carbon and C-O, as the nanodiamonds are heated for a long time. According to the XPS data in Table 1, the sp^2^/sp^3^ ratio slightly decreases as the heating time increases from 2 to 52 h. Here, water and oxygen might be evolved during the long preheating. This could take place at the partially oxidized graphene-like carbon of the shell surrounding the nanodiamond particles, leading to the evolution of traces of oxygen due to the long-term heating. Such removal could be favored in pentagon topological defects due to its higher instability compared with other defects [7,21,22]. The above drive a slight removal of sp^2^-like carbon from the nanodiamond aggregates, resulting in a decrease of the sp^2^/sp^3^ ratio. Furthermore, the wide scan of X-ray photoelectron spectroscopy (XPS) of nanodiamonds before the thermal treatment does not show metallic content, as seen in Figure S3.

**Figure 3:**
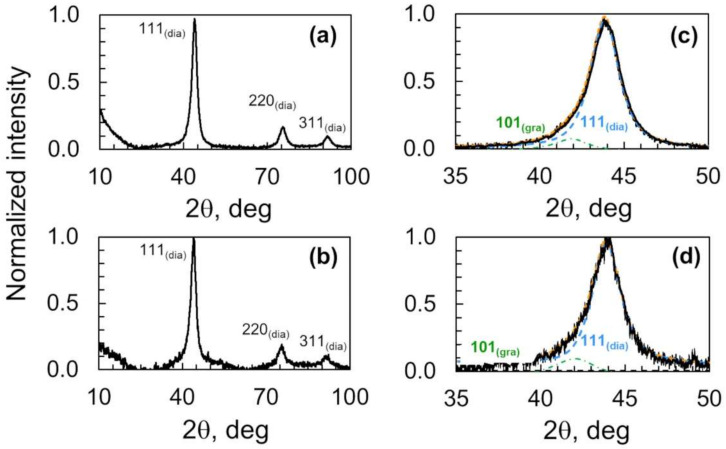
X-ray diffraction patterns of nanodiamonds heated in vacuo at 423 K for (**a**) 2 h and (**b**) 52 h, and corresponding deconvoluted diffraction pattern of the (111) reflection of nanodiamonds heated in vacuo at 423 K for (**c**) 2 h and (**d**) 52 h.

X-ray diffraction patterns of nanodiamonds heated in vacuo at 423 K for 2 h and 52 h are shown in Figure 3. Those profiles show diffraction peaks at 43.8°, 75.4°, and 91.5° that correspond to the reflections from the (111), (220), and (311) lattice planes of nanodiamonds, respectively [7,23]. The most prominent diffraction peak does not change due to preheating for 2 h or 52 h. The X-ray diffraction results confirm the lack of structural change of the nanodiamond core structure surrounded by partially oxidized graphene-like carbons. A closer look at the (111) reflection of nanodiamonds shows the co-presence of the phases sp^3^ and sp^2^ carbon; sp^3^ carbon accounts for at least 90% of the (111) reflection, while the remaining 10% corresponds to the sp^2^ carbon. The prolonged heat treatment (423 K for 52) slightly decreases the sp^2^ carbon phase. Therefore, the sp^2^/sp^3^ ratio was barely modified (0.11 to 0.09), as seen in Table 2.

Table 3 shows the crystallite sizes of nanodiamonds at 43.9°, 75.4°, and 91.5° corresponding to the (111), (220), and (311) facets of nanodiamonds, respectively. The respective crystallite sizes are 5.5, 4.8, and 4.3 nm, which were not modified by the thermal heating at 423 K for 52 h.

### 3.2. Effect of Heating Time on the Pore Structure of Nanodiamond Aggregates

The pore structure of porous adsorbent materials can be described by the adsorption of specific probe molecules. The recommended values of kinetic diameters of probe molecules such as water, argon and nitrogen are 0.27 nm, 0.34 nm, and 0.36 nm [18,19]. The higher adsorption temperature of Ar (87 K) compared with that of the adsorption of nitrogen (77 K) favors the molecular diffusion in the restricted pores. Nevertheless, their size limits their use to describe fine porosity (pores in the range of ultramicropores). Water is a very effective molecular probe to characterize the pore structure where larger molecules such as argon or nitrogen cannot smoothly enter. Water adsorption is carried out at 298 K. Therefore, the diffusion-blocking effect is minimized.

**Figure 4:**
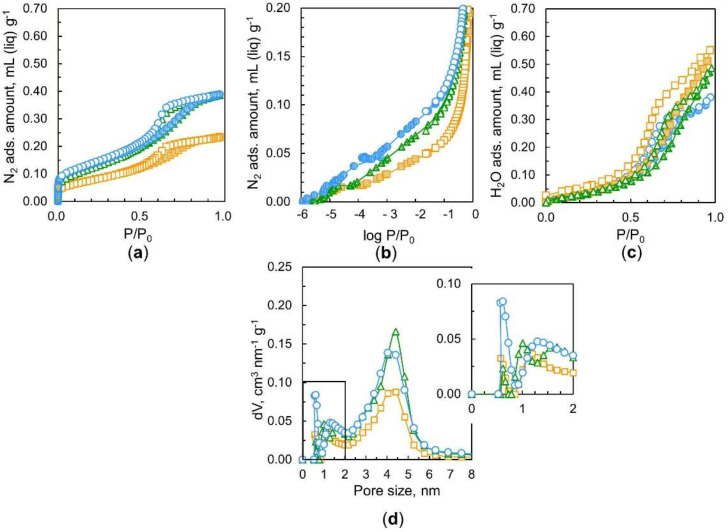
Nitrogen adsorption isotherms at 77 K of nanodiamonds heated in vacuo at 423 K for different times on a (**a**) linear scale and (**b**) logarithmic scale, and (**c**) adsorption isotherms of water at 298 K. (**d**) QS–DFT –derived pore size distributions of nanodiamonds from nitrogen adsorption. The heating times were 2 h (**O**), 10 h (**∆**), and 52 h (**□**). Solid and open symbols in (**a**–**c**) indicate adsorption and desorption branches, respectively.

Our previous works demonstrated that nanodiamond aggregates had some micropores and predominant mesopores of 4.5 nm in average size, resulting in unblocked ultramicropores after outgassing at 423 K for 2 h due to the removal of strongly adsorbed water [7,9]. Consequently, we hypothesized that nanodiamonds outgassed for a longer time should have higher pore availability for nitrogen adsorption. Figure 4a shows the nitrogen adsorption isotherms at 77 K of nanodiamonds treated at 423 K for 2 h, 10 h, and 52 h. Their corresponding logarithmic isotherms are presented in Figure 4b. These typical Type IV adsorption isotherms [24] show considerable adsorption below P/P_0_ = 10^−2^ from adsorption in the small micropores, a gradual increase in adsorption above P/P_0_ = 10^−2^ from adsorption in the large micropores and mesopores, and a subsequently pronounced hysteresis loop from P/P_0_ = 0.5–0.8 due to capillary condensation in the mesopores. The average pore size of the main distribution in the nanodiamond sample is 4.4 nm, as seen in Figure 4d. This pore size expressed by the diameter is similar to those reported in prior literature [7,25]. There was no evident difference in the total nitrogen adsorption between samples preheated at 423 K for 2 h and 10 h, as seen in Figure 4a. However, a clear reduction in nitrogen adsorption after heating for 10 h is observed on a logarithmic scale, as shown in Figure 4b. Therefore, heating for 10 h causes variations in the pore structure. Surprisingly, one can observe a noticeable drop in nitrogen adsorption due to heating for 52 h. Heating for 2 h removes water contained in nanodiamonds. Hence, long-term heating for 52 h should shrink and/or collapse the pores. Figure 4d shows the marked decrease in mesopores at sizes of approximately 4.4 nm. In addition, a pore structure change is observed in the micropore range; the long heating shifts the peak of the supermicropores to a smaller size.

It is well known that water vapor adsorption occurs on microporous carbons on which nitrogen cannot be adsorbed at 77 K owing to differences in the size and nature of the molecules; the molecular size of water is smaller than that of nitrogen, and they can be highly stabilized due to mutual hydrogen bonding even in small pores [26]. Then, the comparison of porosities from nitrogen and water adsorption leads to detailed pore structure analysis. Figure 4c shows linear water adsorption isotherms at 298 K of nanodiamonds preheated in vacuo at 423 K for 2 h, 10 h, and 52 h. The water adsorption isotherms are similar to those of hydrophobic porous carbons, which are IUPAC Type V isotherms [24]. The rising P/P_0_ is similar for the three samples. However, the total adsorption amounts extrapolated to P/P_0_ ≈ 1 are different from each other; longer heating (52 h) yields a larger water adsorption amount. Heating for 52 h provides 0.55 mL g^1^ water adsorption, which is much larger than the amount of the largest nitrogen adsorption in the sample heated for 2 h. The amount of water adsorption in the sample heated for 2 h was almost the same as the amount of nitrogen adsorption under the same heating conditions. Consequently, nitrogen molecules cannot access all the pore spaces because the pore entrances of the samples heated for 10 h and 52 h are blocked. In particular, the most predominant entrance blocking effect is observed for the samples heated for 52 h; the porosity of the sample heated for 52 h with water adsorption is approximately 40% larger than that heated with nitrogen adsorption. Many pores are blocked by nitrogen molecules adsorbed at the pore entrances, whereas water molecules can pass through the entrance barrier. Additionally, nanodiamonds heated for 52 h have large ultramicropore volumes that nitrogen molecules cannot access but where water molecules are accepted. This intensive entrance blocking of only nitrogen adsorption in the long-term heating sample indicates the presence of polar groups near the pore entrances and pore mouth structures.

**Figure 5:**
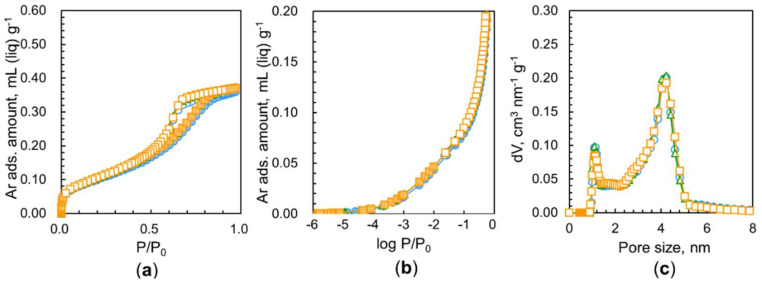
Argon adsorption isotherms at 87 K of nanodiamonds heated in vacuo at 423 K for different times on a (**a**) linear scale and (**b**) logarithmic scale, and (**c**) QS–DFT–derived pore size distributions of nanodiamonds from argon adsorption. The heating times were 2 h (**O**), 10 h (**∆**), and 52 h (**□**). Solid and open symbols in (**a**,**b**) indicate adsorption and desorption branches, respectively.

It is common knowledge that the quadrupole moment of nitrogen interacts with the polar groups in the pore entrances, blocking further nitrogen adsorption at 77 K [24,27]. However, water molecules can form hydrogen bonds with the polar groups at the polar entrances, supporting in-pore diffusion. Thus, comparative pore analysis using the adsorption of nitrogen at 77 K and water at 298 K can provide a detailed pore entrance structure. The IUPAC recommends Ar adsorption at 87 K for the evaluation of small micropores whose pore entrances possess polar groups because Ar has no quadrupole moment. In addition, the adsorption temperature for Ar is 87 K, which is slightly higher than 77 K and favors molecular diffusion in restricted pores. Figure 5 shows Ar adsorption isotherms of samples heated at 423 K for different times. Surprisingly, the Ar adsorption isotherms almost overlap with each other, giving a similar saturated adsorption amount of 0.37 mL g^−1^ at P/P_0_ ≈ 1. This value is close to the nitrogen adsorption amount of the sample heated for 2 h. The pore size distribution shows the presence of micropores and mesopores with diameters of 1.3 nm and 4.4 nm, respectively. Argon adsorption at 87 K in Figure 5 does not show a significant difference between the heating at 423 h for 2 h, 10 h, and 52 h. In contrast, the nitrogen adsorption amount is lowered by preheating exposure. Such a difference in adsorption between these two molecules stems from the pore structure, as stated previously [9]. The structure of pores resulting from preheating in vacuo allows the adsorption of Ar molecules but does not accept nitrogen molecules due to differences in their quadrupole moment.

**Figure 6:**
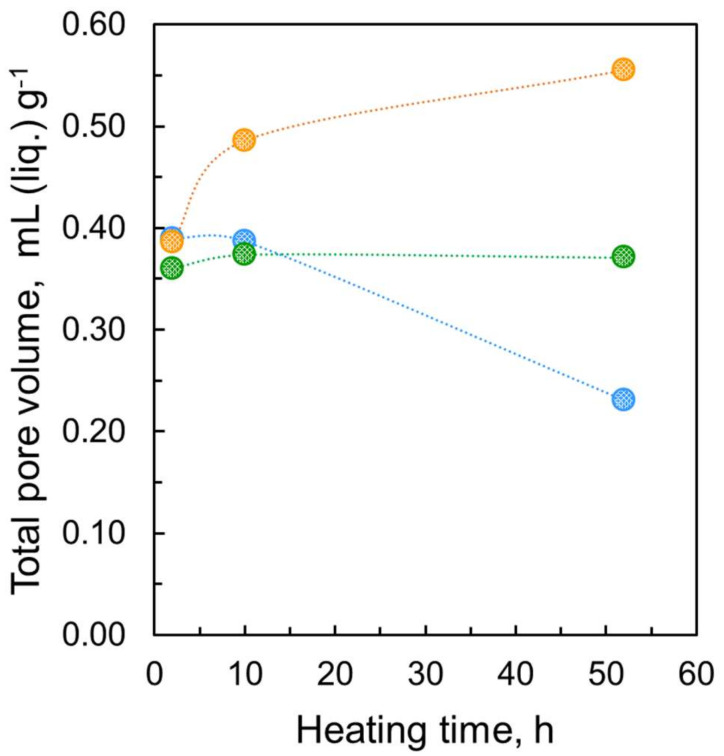
Total pore volume at P/P_0_ ≈ 1 from the adsorption of probe molecules; nitrogen (**O**), water (**O**), and argon (**O**) on nanodiamonds after heating at 423 K for different times. The pore volume expressed in mL g^−^^1^ is evaluated using the liquid nitrogen and water density following the Gurvich rule.

Figure 6 shows a comparison of the total pore volumes evaluated from the adsorption of the probe molecules (water, argon, and nitrogen). The total adsorption amounts extrapolated to P/P_0_ ≈ 1 clearly show that while the total nitrogen uptake decreases over time, water adsorption increases; water adsorption doubles, while nitrogen adsorption is halved at 52 h of heating at 423 K.

Accordingly, all nanodiamond aggregates have a pore network consisting of 1.3 nm micropores and 4.4 nm mesopores irrespective of the heating time at 423 K. These pores are connected to the external surfaces through the pore entrances where the adsorption of nitrogen molecules at 77 K is blocked on the nanodiamond samples heated at 423 K for 10 h and 52 h. The entrance blocking zone must be very narrow, being a representative pore-mouth structure with surface functional groups. However, the samples heated for a long time, such as 52 h, must have narrow micropores among the nanodiamond aggregates that are produced by long-term heating through the evolution of surface oxygen-containing functional groups. Such surface functionalities should be produced under intensive sonication and mechanical milling treatment in aqueous dispersions of nanodiamond particles during the stage of synthesis [11,28,29,30].

Nitrogen and water adsorption isotherms of nanodiamonds heated at 623 K for 2 h, 10 h, and 52 h were similar to those of the treatments at 423 K, as depicted in Figure S4. Nitrogen uptake decreased progressively with heating time due to the removal of water molecules adsorbed on the nanodiamonds. The shape of the hysteresis loop of the water adsorption isotherms of nanodiamonds heated at 623 K for 52 h is similar to IUPAC Type H3 hysteresis loops, although that of nanodiamonds heated at 423 K for 52 h is close to IUPAC Type H2(b) [7,9,10]. The difference is associated with the pore size distribution of the pore entrance. The nanodiamonds heated at 423 K for 52 h must have a wider size distribution at the pore entrances than those at 623 K. Heating at 623 K for 52 h deprives the sample of more surface oxygen than heating at 623 K for 2 h, according to the XPS analyses shown in Figure S2 and Table S1. Heating for 52 h induces a higher P/P_0_ shift in the rising pressure of the water adsorption isotherm. The water adsorption behavior of the heated nanodiamonds is quite different from the adsorption of nitrogen behavior; therefore, comparative analysis using the adsorption of nitrogen and water is indispensable for understanding the pore structure of nanodiamonds.

**Figure 7:**
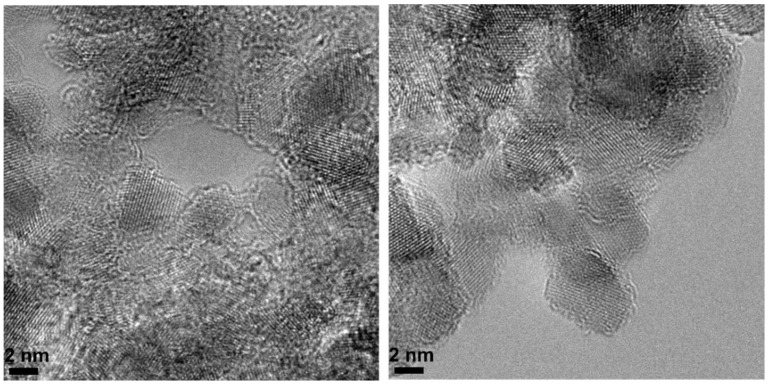
HRTEM images of nonthermally treated detonation nanodiamonds.

HRTEM shows direct images of the structural organization of the nanodiamond particles. The compact arrangement of detonation nanodiamonds leaves inter-particle pores whose dimensions are in the range of 2–6 nm, as seen in Figure 7. Some pores might be connected to others or the exterior through pore mouth entrances, allowing the selective adsorption of probe molecules.

Table 4 summarizes the main components of the proposed pore structure and adsorption mechanism on long-term treated nanodiamonds at 423 K based on the experimental results presented in this manuscript.

## 4. Discussion

A detonation nanodiamond particle is partially wrapped by graphene-like carbons that are not completely oxidized. Preceding studies on water adsorption elucidated the importance of the water molecules strongly bound in the interstices between the diamond core and graphene-like carbon [6,7,9]. As the nanodiamonds are treated by intensive disaggregation treatment, such as high-power sonication and stirred-mediated milling, to obtain their stable dispersion, the primary nanodiamond particles must present a slight amount of oxygenated functional groups. Dried nanodiamond particles of hard hydrogels of nanodiamonds should form hydrogen bond-mediated agglomeration structures. The presence of the oxygen functional groups on the nanodiamond shell allows for hydrogen bonding with other -OH groups, hydrogen atoms, and other molecules. Hydrogen bonding occurs through surface oxygen functionalities, which can be located at the pore mouth structures of micropores and mesopores; thus, the ultramicropores are effectively closed by the surface oxygen-containing functionalities. Here, only water molecules can access the ultramicropores after the removal of those groups. Long-term vacuum dehydration at 423 K opens the pore-mouth gate and ultramicropores. The size of the pore-mouth gate must be in the range of 0.4 to 0.7 nm. Both nitrogen and argon can access the pores. However, nitrogen molecules strongly adsorb with quadrupole-associated interactions at the pore mouths, blocking further nitrogen adsorption inside the pores. As a result, long-term dehydration decreases the porosity by nitrogen adsorption and increases it by water adsorption. Therefore, mildly oxidized detonation nanodiamond particles can show unique molecular sieving behavior.

The properties of nanodiamond aggregates are associated with the properties of their primary particles, such as surface chemistry, purity, structure, and pore configuration, in nanodiamond assembly. Detonation nanodiamonds might contain metallic and non-metallic impurities derived from the synthesis materials, detonation chamber, and the use of acids, alkalis, or complex reagents for purification [31]. Furthermore, the surface of nanodiamonds can be modified by the sorption of ions [32]. The presence of non-carbon impurities and ions impact the physical and surface chemical properties of the adsorbent material, which might influence the adsorption properties. In the present study, water and nitrogen adsorption are not influenced by ions, since the water adsorption and nitrogen adsorption are performed at low temperatures (298 K and 77 K, respectively). In addition, the wide scan of XPS of nonthermally treated nanodiamonds does not show metallic content, as shown in Figure S3. Therefore, the interaction of ions and metallic ions with water was ruled out. Aggregates of tightly conglomerated primary nanodiamond particles [30] are governed by hydrogen bonding interactions [29,33] and driven by oxygen-containing surface groups, such as hydroxyl, carboxyl, carbonyl, ketone groups, etc. Heating nanodiamonds at 423 K for 2 h desorbs water, while heating for 10 h induces a pore structure change in addition to the desorption of water. A more exhaustive heating in vacuo (423 K for 52 h) causes shrinkage/collapse of the pores, resulting in low nitrogen adsorption. Heating at 423 K in an inert atmosphere induces the removal of water present in the nanodiamond aggregates, resulting in spontaneous self-assembly [34,35]. Furthermore, treating nanodiamonds at temperatures of 423 K for a long time partially removes surface oxygen-containing functional groups. XPS data show a slight decrease in the O/C ratio as the heating time increases. The above conditions are responsible for a new highly aggregated structure of nanodiamonds with a different configuration of pores, disorder aggregation and order domains [36], and characteristic pore-mouth structures. It is well known that the removal of hydroxyls by heating results in the formation of ether groups due to dehydration and that surface ether groups also enhance the tight aggregation of agglomerated nanodiamonds [37].

Recently, researchers have reported the use of detonation nanodiamonds for the reversible sorption of metal cations [32]. New studies of molecular dynamic (MD) simulations have included the presence of ions adsorbed on the surface of nanodiamonds exposed to aqueous salts solutions to describe the dynamics of water around detonation nanodiamonds. Here, positively charged nanodiamonds (e.g., ND-H or ND-NH_2_) have affinity to anions, and negatively charged nanodiamonds (e.g., ND-COOH) have affinity to cations. The presence of the counterions influences the water motion nearby the nanodiamond [38]. As a result, different degrees of water affinity could be controlled.

The water adsorption process on the nanodiamond surface has been studied through simulations to understand the water affinity upon the surface chemistry. In a particular study, the simulation of water adsorption on nanodiamonds surrounded by graphitic carbon has demonstrated that charged polar groups determine the preferred orientation of the interfacial water [39]. In addition, according to density functional theory (DFT) calculations, the orientation of its -OH bonds of water molecules and the dipole moment depend on the surface nature of nanodiamonds; in the regions without graphitic carbon, it is expected that one of the -OH bonds in water will interact favorably with the diamond surface including carbon with dangling bonds, but both -OH are away from a fully hydrogenated surface [40]. During the adsorption of water within nanodiamond aggregates, it is expected that water first adsorbs on the surface of the nanodiamonds, and subsequently, more water would accumulate within the pores due to hydrogen bonding between water molecules.

As mentioned earlier, the present manuscript proves that the removal of water and oxygen-containing surface groups (obtained by exhaustive heating in vacuo) triggers the opening of the pore-mouth gate and the availability of more ultramicropores that promote higher water adsorption. Such behavior sets a precedent for the finer control of water adsorption using pore availability in nanodiamond aggregates.

## 5. Conclusions

Nanodiamonds are composed of aggregated nanodiamond particles that have mesopores and micropores with diameters of 4.4 and 1.3 nm, respectively, as well as scarce ultramicropores whose width is 0.4 nm; these pores adsorb water, nitrogen, and argon of approximately 0.38 mL g^−1^ at P/P_0_ ≈ 1. The extent of such adsorption is controlled by heat treatments. Long-term treatment at 423 K for 52 h in vacuo dehydrates the aggregates, partially removes surface oxygen groups, opens the pore mouths, and unblocks ultramicropores, allowing 40% more water adsorption than that on nanodiamonds that are only heated for a short time (423 K for 2 h). In contrast, a long heating treatment in vacuo (423 K for 52 h) decreases the porosity evaluated by nitrogen adsorption by 40% due to the shrinkage of pores and adsorption of nitrogen molecules at the pore-mouth entrances; such adsorption obstructs further nitrogen adsorption. As a result, the unique molecular sieving properties of mildly oxidized detonation nanodiamonds are dependent on the pore structure defined by the aggregation of nanodiamond particles that are influenced by the hydration level and the presence of the newly described characteristic pore-mouth entrances of mesopores.

## Figures and Tables

**Table 1:** Constituents of C 1 s (at %) from XPS analyses of nonheated and heated nanodiamonds in vacuo.

C 1 s Components	Nonheated ND		423 K for 2 h		423 K for 52 h	
	Binding Energy, eV	% Area	Binding Energy, eV	% Area	Binding Energy, eV	% Area
C sp^2^	284.3	9.4	284.3	8.5	284.3	6.7
C sp^3^	285.2	54.2	285.1	56.4	285.1	57.2
C-H, C-N, C-OH	286.2	29.8	286.2	30.1	286.2	31.1
C-O	287.3	6.6	287.5	5.0	287.5	5.0
sp^2^/sp^3^ ratio:	0.17		0.15		0.12	

**Table 2:** Contents of sp^2^ and sp^3^ phases of nanodiamonds heated in vacuo at 423 K for 2 h and 52 h from the (111) reflection of nanodiamonds obtained by X-ray diffraction.

Sample Name	Phase Name	Crystalline Plane	Peak Position 2θ, deg	Peak Area 2θ, deg	% Area	Ratio sp^2^/sp^3^
ND-2 h	Graphite (sp^2^)	(101)	41.9	0.27	0.10	0.11
	Diamond (sp^3^)	(111)	43.9	2.39	0.90	
ND-52 h	Graphite (sp^2^)	(101)	41.9	0.16	0.08	0.09
	Diamond (sp^3^)	(111)	43.9	1.75	0.92	

**Table 3:** Crystallite sizes of the different facets of nanodiamonds (111), (220), and (311) before and after thermal treatment from X-ray diffraction analysis.

Sample Name	Phase Name	Crystalline Plane	Peak Position 2θ, deg	FWHM (as 2θ, deg)	Crystallite Size, nm
ND-2 h	Diamond	(111)	43.9	2.07	5.5
	Diamond	(220)	75.4	2.56	4.8
	Diamond	(311)	91.5	2.66	4.3
ND-52 h	Diamond	(111)	43.9	2.03	5.6
	Diamond	(220)	75.3	2.48	5.0
	Diamond	(311)	91.6	2.63	4.3

**Table 4:** Main components and facts of the pore model of long-term heated nanodiamonds.

Component	Description
Pore mouth structure	Quadrupole moment of nitrogen interacts with the local electric fields coming from surface oxygens after dehydration. Nitrogen adsorbed at the pore mouth suppresses further nitrogen adsorption. Long-term dehydration partially distorts the pore mouth, decreasing the nitrogen adsorption. Argon molecules are accessible.
Mesopores	Mesopores are connected by the pore mouth. The adsorption in mesopores is controlled by the pore mouth.
Ultramicropores	Small ultramicropores where only water molecules are accessible. Their size is probably < 0.4 nm in diameter. The interstices between the nanodiamond core and the partially oxidized graphene-like carbon shell can contribute to preferential water adsorption.
An illustration of the pore mouth model of nanodiamonds is presented as the graphical abstract of the present manuscript.	

## Data Availability

The data that support the findings of this study are available from the corresponding author upon reasonable request.

## Supplementary Materials

The following are available online at https://www.mdpi.com/article/10.3390/nano11112772/s1. Figure S1: Comparison of thermogravimetric (TG) profiles; TG differential profiles of nonheated and heated nanodiamonds at 423 K and 623 K for 2 h and 52 h under an argon atmosphere. Figure S2: XPS analyses of nonheated and heated nanodiamonds at 423 K and 623 K for 2 h and 52 h. (a) Atomic contents (at %) of the C 1s, O 1s, and N 1s core levels, and (b) deconvolution of the high-resolution C 1s spectra, Table S1: Constituents of C 1s (at %) from XPS analyses of nonheated and heated nanodiamonds in vacuo. Figure S3: XPS wide scan of nonheated nanodiamonds. Figure S4: Nitrogen adsorption isotherms at 77 K of nanodiamonds heated in vacuo at 623 K on a linear scale and logarithmic scale; adsorption isotherm of water at 298 K; QS–DFT–derived pore size distributions from nitrogen adsorption of nanodiamonds. The heating times were 2 h, 10 h, and 52 h.

## References

[1] Dufour A., Celzard A., Fierro V., Broust F., Courson C., Zoulalian A., Rouzaud J. (2015). Catalytic conversion of methane over a biomass char for hydrogen production: Deactivation and regeneration by steam gasification. Appl. Catal. A Gen..

[2] Grosso-Giordano N.A., Schroeder C., Xu L., Solovyov A., Small D.W., Koller H., Zones S.I., Katz A. (2021). Characterization of a Molecule Partially Confined at the Pore Mouth of a Zeotype. Angew. Chem. Int. Ed..

[3] Lanin S.N., Platonova S.A., Vinogradov A.E., Lanina K.S., Nesterenko E.P., Nesterenko P. (2019). Comparative study of different polar adsorbents for adsorption of water soluble vitamins. Adsorption.

[4] Pacelli S., Maloney R., Chakravarti A.R., Whitlow J., Basu S., Modaresi S., Gehrke S., Paul A. (2017). Controlling Adult Stem Cell Behavior Using Nanodiamond-Reinforced Hydrogel: Implication in Bone Regeneration Therapy. Sci. Rep..

[5] Gusain R., Kumar N., Ray S.S. (2020). Recent advances in carbon nanomaterial-based adsorbents for water purification. Coord. Chem. Rev..

[6] Jiang T., Xu K. (1995). FTIR study of ultradispersed diamond powder synthesized by explosive detonation. Carbon.

[7] Pina-Salazar E.-Z., Urita K., Hayashi T., Futamura R., Vallejos-Burgos F., Włoch J., Kowalczyk P., Wiśniewski M., Sakai T., Moriguchi I. (2017). Water Adsorption Property of Hierarchically Nanoporous Detonation Nanodiamonds. Langmuir.

[8] Petit T., Girard H.A., Trouvé A., Batonneau-Gener I., Bergonzo P., Arnault J.-C. (2013). Surface transfer doping can mediate both colloidal stability and self-assembly of nanodiamonds. Nanoscale.

[9] Piña-Salazar E.-Z., Kukobat R., Futamura R., Hayashi T., Toshio S., Ōsawa E., Kaneko K. (2018). Water-selective adsorption sites on detonation nanodiamonds. Carbon.

[10] Ji S., Jiang T., Xu K., Li S. (1998). FTIR study of the adsorption of water on ultradispersed diamond powder surface. Appl. Surf. Sci..

[11] Osawa E., Akasaka T., Wudl F., Nagase S. (2010). Chemistry of Single-Nano Diamond Particles. Chemistry of Nanocarbons.

[12] Hattori Y., Kaneko K., Ohba T. (2013). Adsorption Properties. Comprehensive Inorganic Chemistry II.

[13] Bi H., Yin K., Xie X., Ji J., Wan S., Sun L., Terrones M., Dresselhaus M.S. (2013). Ultrahigh humidity sensitivity of graphene oxide. Sci. Rep..

[14] Yao Y., Chen X., Ma W., Ling W. (2014). Quartz crystal microbalance humidity sensors based on nanodiamond sensing films. IEEE Trans. Nanotechnol..

[15] Piña-Salazar E.Z., Sakai T., Ōsawa E., Futamura R., Kaneko K. (2019). Unusual hygroscopic nature of nanodiamonds in comparison with well-known porous materials. J. Colloid Interface Sci..

[16] Piña-Salazar E.-Z., Sagisaka K., Hattori Y., Sakai T., Futamura R., Ōsawa E., Kaneko K. (2019). Electrical conductivity changes of water-adsorbed nanodiamonds with thermal treatment. Chem. Phys. Lett. X.

[17] Yu X., Chen X., Ding X., Chen X., Yu X., Zhao X. (2019). High-sensitivity and low-hysteresis humidity sensor based on hydrothermally reduced graphene oxide/nanodiamond. Sens. Actuators B Chem..

[18] Sing K.S.W., Williams R.T. (2004). The Use of Molecular Probes for the Characterization of Nanoporous Adsorbents. Part. Part. Syst. Charact..

[19] Kobori R., Ohba T., Suzuki T., Iiyama T., Ozeki S., Inagaki M., Nakamura A., Kawai M., Kanoh H., Kaneko K. (2009). Fine pore mouth structure of molecular sieve carbon with GCMC-assisted supercritical gas adsorption analysis. Adsorption.

[20] Stehlik S., Glatzel T., Pichot V., Pawlak R., Meyer E., Spitzer D., Rezek B. (2016). Water interaction with hydrogenated and oxidized detonation nanodiamonds—Microscopic and spectroscopic analyses. Diam. Relat. Mater..

[21] Fujimori T. (2012). Selective probe of the morphology and local vibrations at carbon nanoasperities. J. Chem. Phys..

[22] Pina-Salazar E.Z., Kaneko K. (2015). Adsorption of water vapor on mesoporosity-controlled singe wall carbon nanohorn. Colloids Interface Sci. Commun..

[23] Shenderova O.A., Vlasov I.I., Turner S., Van Tendeloo G., Orlinskii S.B., Shiryaev A.A., Khomich A.A., Sulyanov S.N., Jelezko F., Wrachtrup J. (2011). Nitrogen Control in Nanodiamond Produced by Detonation Shock-Wave-Assisted Synthesis. J. Phys. Chem. C.

[24] Thommes M., Kaneko K., Neimark A.V., Olivier J.P., Rodriguez-Reinoso F., Rouquerol J., Sing K.S.W. (2015). Physisorption of gases, with special reference to the evaluation of surface area and pore size distribution (IUPAC Technical Report). Pure Appl. Chem..

[25] Kaneko K. (1994). Determination of pore size and pore size distribution 1. Adsorbents and catalysts. J. Membr. Sci..

[26] Setoyama N., Ruike M., Kasu T., Suzuki T., Kaneko K. (1993). Surface Characterization of Microporous Solids with He Adsorption and Small Angle X-ray Scattering. Langmuir.

[27] Vallejos-Burgos F., Coudert F.-X., Kaneko K. (2018). Air separation with graphene mediated by nanowindow-rim concerted motion. Nat. Commun..

[28] Ozawa M., Inaguma M., Takahashi M., Kataoka F., Krüger A., Osawa E. (2007). Preparation and behavior of brownish, clear nanodiamond colloids. Adv. Mater..

[29] Ōsawa E., Ho D., Huang H., Korobov M.V., Rozhkova N.N. (2009). Consequences of strong and diverse electrostatic potential fields on the surface of detonation nanodiamond particles. Diam. Relat. Mater..

[30] Krüger A., Kataoka F., Ozawa M., Fujino T., Suzuki Y., Aleksenskii A., Vul’ A.Y., Ōsawa E. (2005). Unusually tight aggregation in detonation nanodiamond: Identification and disintegration. Carbon.

[31] Mitev D.P., Townsend A.T., Paull B., Nesterenko P.N. (2013). Direct sector field ICP-MS determination of metal impurities in detonation nanodiamond. Carbon.

[32] Volkov D.S., Krivoshein P.K., Mikheev I.V., Proskurnin M.A. (2020). Pristine detonation nanodiamonds as regenerable adsorbents for metal cations. Diam. Relat. Mater..

[33] Pentecost A., Gour S., Mochalin V., Knoke I., Gogotsi Y. (2010). Deaggregation of nanodiamond powders using salt- and sugar-assisted milling. ACS Appl. Mater. Interfaces.

[34] Kowalczyk P., Piña-Salazar E.-Z., Kirkensgaard J.J.K., Terzyk A.P., Futamura R., Hayashi T., Ōsawa E., Kaneko K., Ciach A. (2020). Reconstructing the fractal clusters of detonation nanodiamonds from small-angle X-ray scattering. Carbon.

[35] Chang S.L.Y., Reineck P., Williams D., Bryant G., Opletal G., El-Demrdash S.A., Chiu P.-L., Ōsawa E., Barnard A.S., Dwyer C. (2020). Dynamic self-assembly of detonation nanodiamond in water. Nanoscale.

[36] Opletal G., Chang S.L., Barnard A.S. (2020). Simulating facet-dependent aggregation and assembly of distributions of polyhedral nanoparticles. Nanoscale.

[37] Katsiev K., Solovyeva V., Mahfouz R., Abou-Hamad E., Peng W., Idriss H., Kirmani A.R. (2021). Fresh insights into detonation nanodiamond aggregation: An X-ray photoelectron spectroscopy, thermogravimetric analysis, and nuclear magnetic resonance study. Eng. Rep..

[38] Saberi-Movahed F., Brenner D.W. (2012). Impacts of surface chemistry and adsorbed ions on dynamics of water around detonation nanodiamond in aqueous salt solutions. arXiv.

[39] Saberi-Movahed F., Brenner D.W. (2021). What drives adsorption of ions on surface of nanodiamonds in aqueous solutions?. arXiv.

[40] Manelli O., Corni S., Righi M.C. (2010). Water Adsorption on Native and Hydrogenated Diamond (001) Surfaces. J. Phys. Chem. C.

