# Supplementary material for: Pore-Mouth Structure of Highly Agglomerated Detonation Nanodiamonds
Source: Nanomaterials (Basel). 2021 Oct 20;11(11):2772. doi: 10.3390/nano11112772 (PMC8618090; doi:10.3390/nano11112772)
Supplement: Supplementary file 1 [file nanomaterials-11-02772-s001.zip › nanomaterials-1369003-supplementary.pdf]

Supplementary Material

Pore-Mouth Structure of Highly Agglomerated Detonation Nanodiamonds

Elda Zoraida Piña-Salazar [1], Kento Sagisaka [2], Takuya Hayashi [1], Yoshiyuki Hattori [1,2], Toshio Sakai [3], Eiji Ōsawa [4] and Katsumi Kaneko [1,*]

[1] Research Initiative for Supra-Materials, Interdisciplinary Cluster for Cutting Edge Research, Shinshu University, Nagano 380-8553, Japan; eldazps@shinshu-u.ac.jp (E.Z.P.-S.); hayashi@endomoribu.shinshu-u.ac.jp (T.H.); hattoriy@shinshu-u.ac.jp (Y.H.)

[2] Division of Chemistry and Materials, Faculty of Textile Science and Technology, Shinshu University, Ueda 386-8567, Japan; 16st302j@shinshu-u.ac.jp (K.S.); hattoriy@shinshu-u.ac.jp (Y.H.)

[3] Department of Materials Chemistry, Faculty of Engineering, Shinshu University, Nagano 380-8553, Japan; tsakai@shinshu-u.ac.jp

[4] Nano-Carbon Research Institute, Ltd., Ueda 386-8567, Japan; osawa@nano-carbon.jp

* Correspondence: kkaneko@shinshu-u.ac.jp

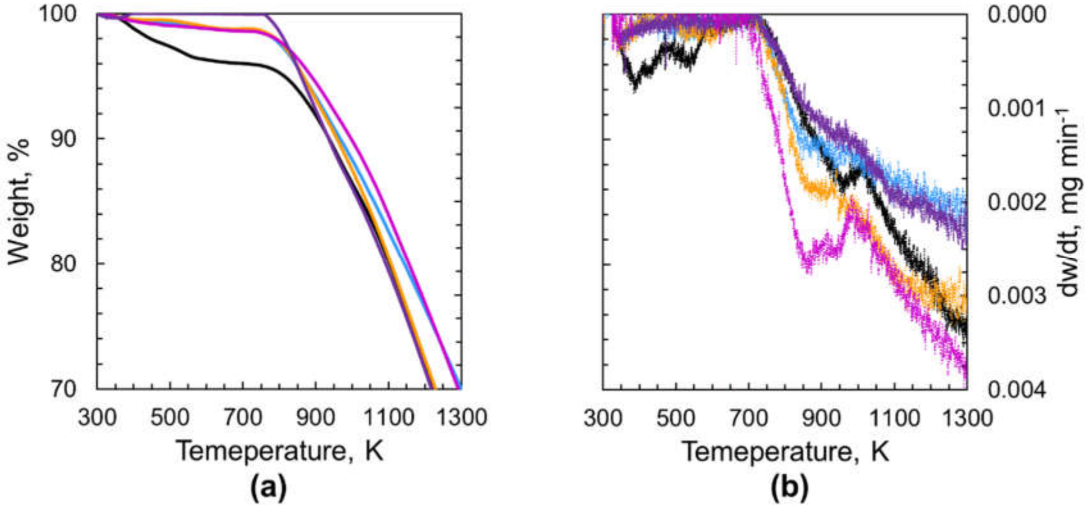

Figure S1. Comparison of (**a**) thermogravimetric (TG) and (**b**) TG differential profiles of nonheated nanodiamonds (black line **-**) and nanodiamonds heated at 423 K for 2 h (light blue line -) and 52 h (orange line -); and at 623 K for 2 h (purple line -) and 52 h (pink line -) under an argon atmosphere (Ar 100 mL min^{-1}).

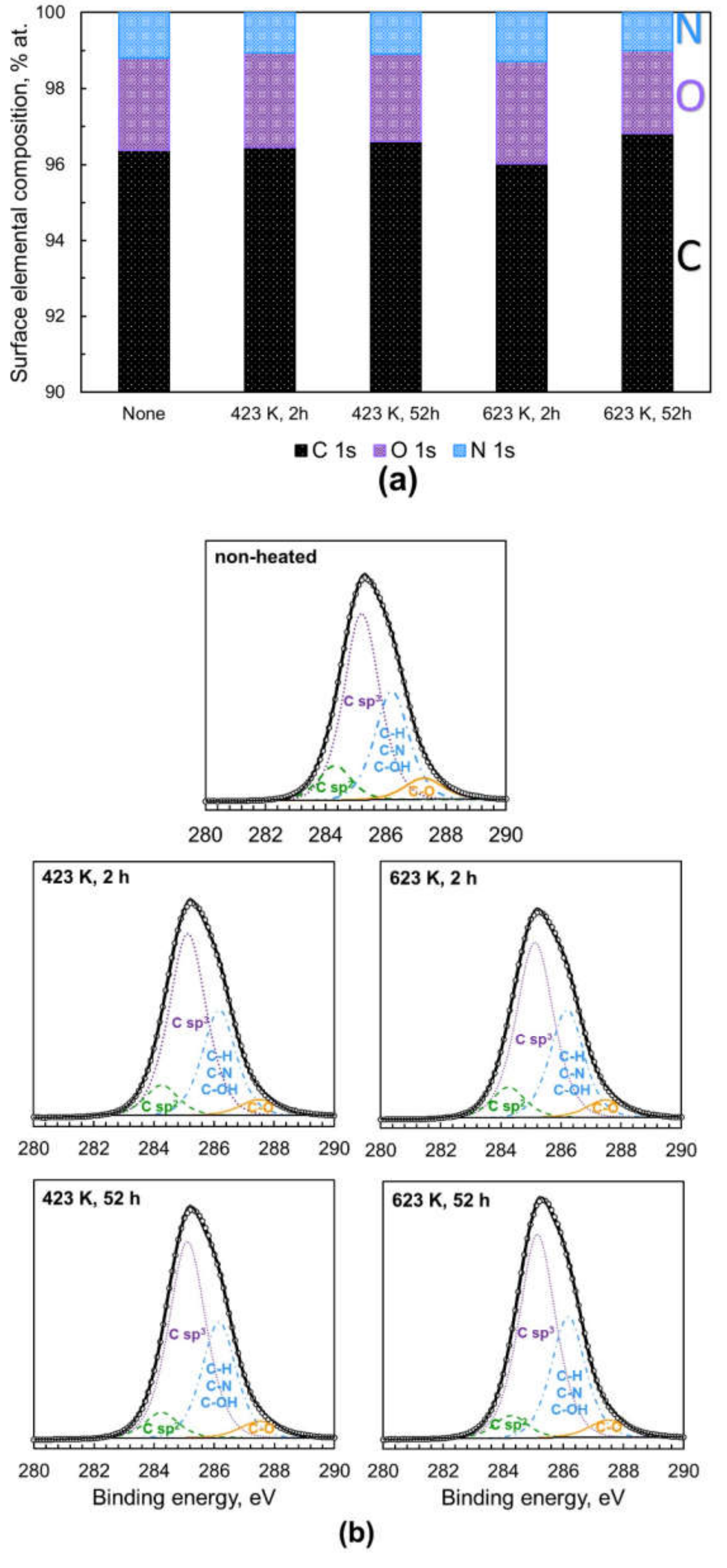

Figure S2. XPS analyses nonheated and heated nanodiamonds at 423 K and 623 K for 2 h and 52 h. (**a**) atomic contents (at. %) of C 1s, O 1s, and N 1s core levels, and (**b**) deconvolution of the high-resolution C 1s spectra; black bold line, open circles, and colored lines depict the experimental, fitted and deconvoluted spectra, respectively.

Table S1. Constituents of C 1s (at. %) from XPS analyses of nonheated and heated nanodiamonds in *vacuo*.

C 1s components	nonheated ND		**423 K** for **2 h**		**423 K** for **52 h**		**623 K** for **2 h**		**623 K** for **52 h**	
	Position	%Area	Position	%Area	Position	%Area	Position	%Area	Position	%Area
C sp^2	284.3	9.4	284.3	8.5	284.3	6.7	284.3	8.6	284.3	5.8
C sp^3	285.2	54.2	285.1	56.4	285.1	57.2	285.1	54.8	285.1	57.6
C-H, C-N, C-OH	286.2	29.8	286.2	30.1	286.2	31.1	286.2	31.0	286.2	31.5
C-O	287.3	6.6	287.5	5.0	287.5	5.0	287.5	5.6	287.5	5.1
sp^2/sp^3 ratio:	0.17		0.15		0.12		0.16		0.10	

Fitting accuracy: >94%, G-L (60-40), FWHM 1.4 + 1.0 eV

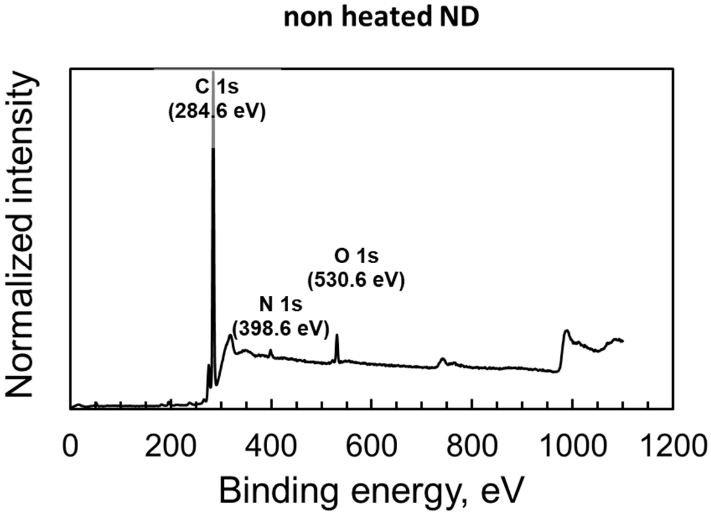

Figure S3. XPS wide scan of nonheated nanodiamonds. The main constituents C 1s, O 1s and N1 s are plotted in bold. The plots corresponding to nanodiamonds heated at 423 K and 623 K for 2 and 52 h are not shown.

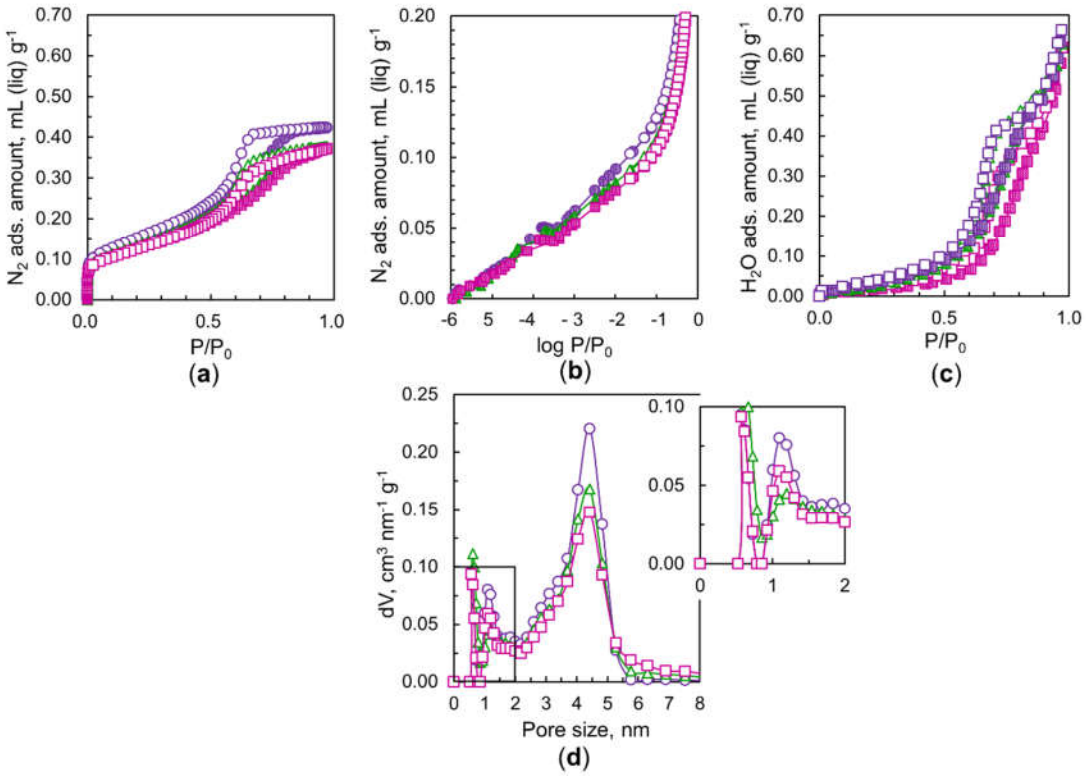

Figure S4. Nitrogen adsorption isotherms at 77 K of nanodiamonds heated in *vacuo* at 623 K for different times on (**a**) linear scale and (**b**) logarithmic scale, and (**c**) adsorption isotherms of water at 298 K. (**d**) QS-DFT-derived pore size distributions of nanodiamonds from nitrogen adsorption. The heating times were 2 h (O), 10 h (Δ) and 52 h (□). Solid and open symbols in (a), (b) and (c) indicate adsorption and desorption branches, respectively.
